# Highlighting the positive aspects of being a PhD student

**DOI:** 10.7554/eLife.81075

**Published:** 2022-07-26

**Authors:** Camille Bernery, Léo Lusardi, Clara Marino, Martin Philippe-Lesaffre, Elena Angulo, Elsa Bonnaud, Loreleï Guéry, Eléna Manfrini, Anna Turbelin, Céline Albert, Ugo Arbieu, Franck Courchamp

**Affiliations:** 1 Laboratoire Écologie Systématique Évolution, Université Paris-Saclay, CNRS and AgroParisTech Orsay France; 2 Estación Biológica de Doñana, CSIC Sevilla Spain; 3 UMR Plant Health Institute of Montpellier, CIRAD and INRAE Montpellier France; 4 Senckenberg Biodiversity and Climate Research Centre Frankfurt am Main Germany; 5 Smithsonian Conservation Biology Institute Front Royal United States

**Keywords:** research culture, graduate school, early-career researchers, doctorate, well-being, None

## Abstract

Articles about doing a PhD tend to focus on the difficulties faced by research students. Here we argue that the scientific community should also highlight the positive elements of the PhD experience.

## Introduction

Doing a PhD can be both demanding and rewarding. In addition to overcoming the scientific and intellectual challenges involved in doing original research, a PhD student may also have to deal with financial difficulties, an unhealthy work-life balance, or resulting concerns about their mental health (Woolston, 2017; Auerbach et al., 2018; Oswalt et al., 2020; Evans et al., 2018). Despite all this, most PhD students seem satisfied with their decision to do a PhD, mostly because they work in stimulating environments with a high degree of independence and good supervision (Pommier et al., 2022; Woolston, 2017).

**Figure 1.**
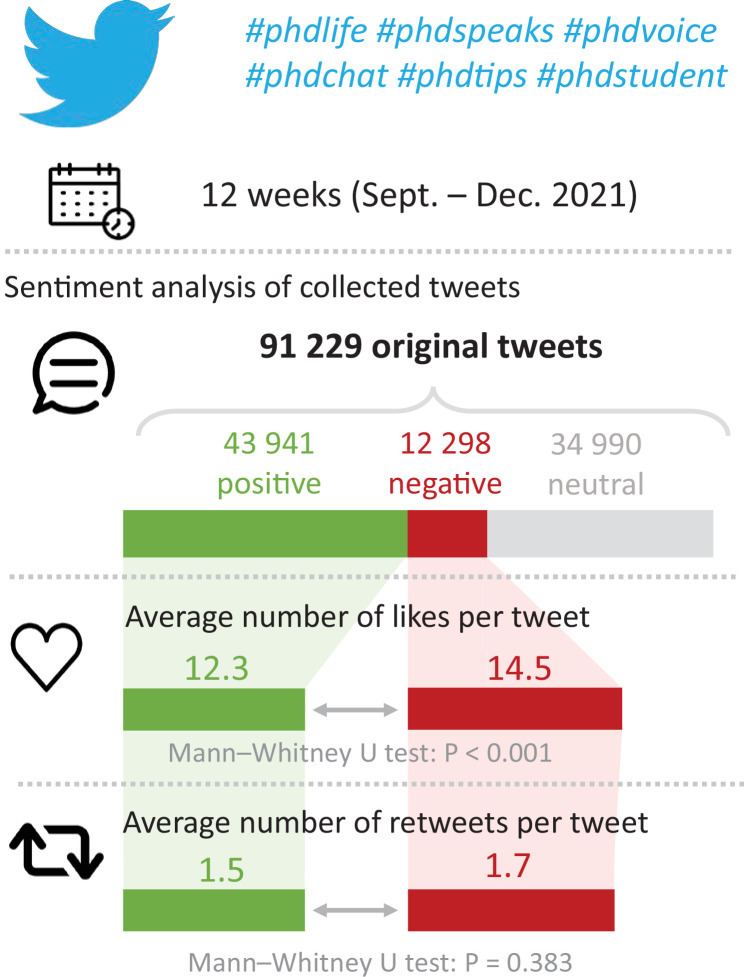
Sentiment analysis of tweets about the PhD experience. We retrieved all tweets posted in the English language during 12 consecutive weeks, from September to December 2021, that contained any of the following six hashtags: #phdlife, #phdspeaks, #phdvoice, #phdchat, #phdtips, #phdstudent. We then measured the sentiment (positive, negative or neutral) associated with each original tweet (excluding retweets). Of the 91 229 tweets we retrieved, 43,941 were positive, 12,298 were negative, and 34,990 were neutral. Mann-Whitney U tests were performed to compare the average number of likes and retweets of positive versus negative tweets. Negative tweets received significantly more likes than positive tweets (14.5 vs 12.3; *P*<0.001); negative tweets were also retweeted more than positive tweets but the difference was not significant (1.7 vs 1.5; *P*=0.383). The Twitter API and the “rtweet” R package (cran.r-project.org/web/packages/rtweet/vignettes/intro.html) were used to retrieve the tweets; the “syuzhet” R package (rdrr.io/cran/syuzhet/) and the Bing lexicon (Liu, 2012) were used for the sentiment analysis; all analyses were performed with R software (R Development Core Team, 2021).

Paradoxically, however, the fact that most PhD students are positive about doing a PhD is not always apparent to the outside world. For example, the present authors recently analysed more than 90,000 tweets about the PhD experience: almost half of the tweets were positive, and less than a sixth were negative, yet the negative tweets received more likes and retweets (Figure 1). What can be done to counter such misleading and negative impressions? In this article we – a group of PhD students, postdocs and permanent academics – highlight the positive elements of doing a PhD in order to present a more balanced view of the whole PhD experience. We also make recommendations to maintain a positive momentum throughout the PhD. Although these ideas and recommendations are based on our experiences as researchers in ecology working in Europe, we feel that most of the points we make also apply in other disciplines and places.

## Three benefits of doing a PhD

**Figure 2.**
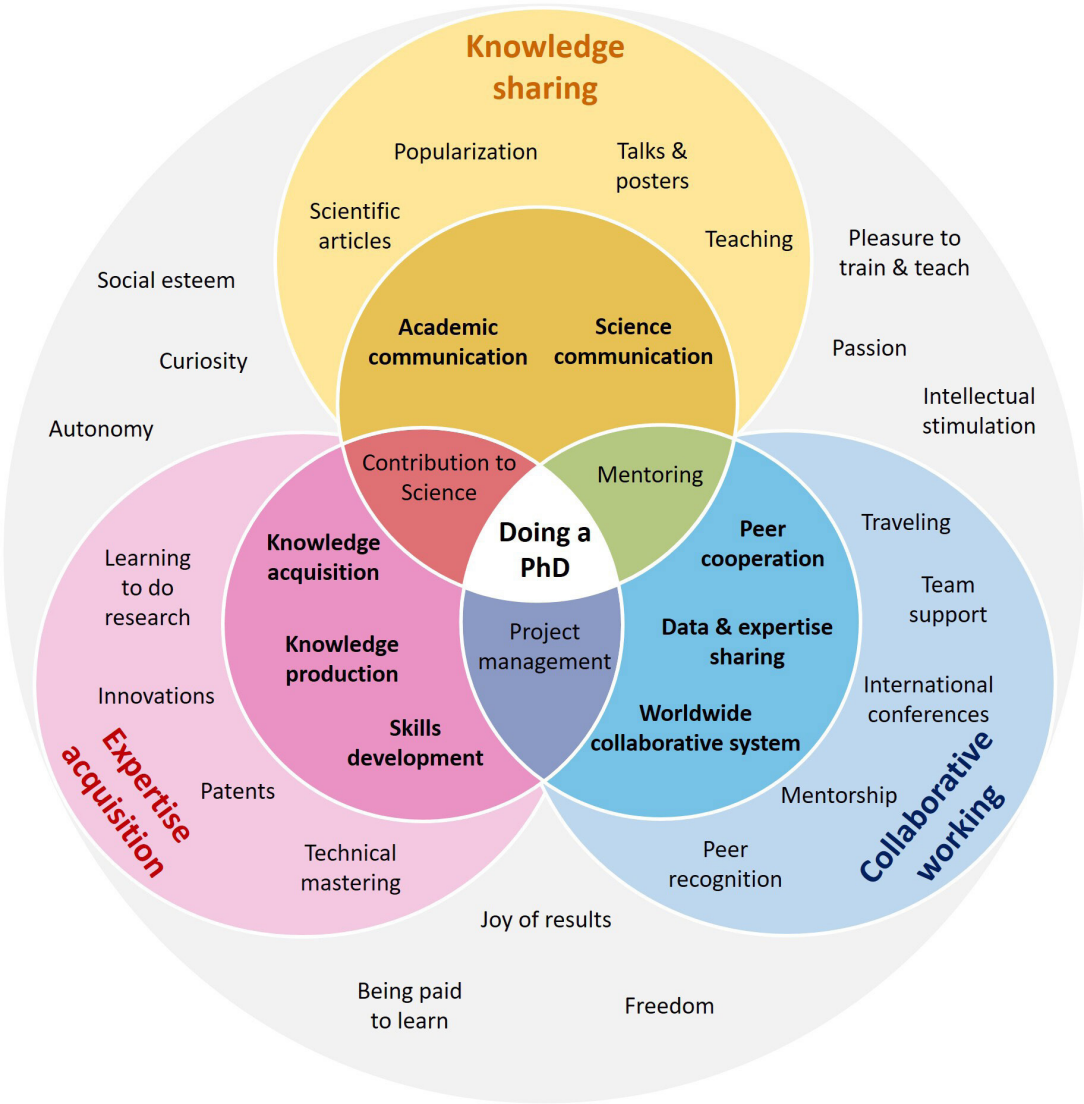
The positive aspects of doing a PhD. The three primary benefits of doing a PhD are acquiring expertise (pink circle), learning to work in a collaborative environment (blue), and developing communication skills for sharing knowledge (yellow). For each benefit, general aspects that apply to almost all doctoral students are shown in bold type in the small circle, and specific aspects that depend on, for example, the student’s supervisor or field of research are shown in plain type in the large circle. The large grey area contains more abstract and subjective ideas that are not discussed in the main text. It should be noted that this figure is conceptual, and that the aspects and ideas in it could be grouped in other, equally valid, ways.

There are two primary outputs from a PhD: new skills and expertise for the graduate, and new knowledge for the wider world. In this article we focus on the former and discuss the three main benefits of doing a PhD for the individual: (i) the development of specific skills to become an expert; (ii) the ability to work in a collaborative environment; (iii) improved communication skills while sharing knowledge (Figure 2). For each of these benefits we discuss both general aspects that apply to most doctoral students, and specific aspects that depend on the student’s supervisor, field of research, location and other factors.

### Becoming an expert

Throughout a doctoral project, a PhD student will develop many of the skills needed to grow into an independent researcher, while also developing expertise in a given field. In addition to learning a great deal about their own field – and adding knowledge to it – a PhD student will learn how to perform a variety of tasks, and thus acquire new transferable skills. These will include autonomy, critical thinking, organization and planning, resilience, and the ability to design, lead and carry out projects. Furthermore, unlike postdocs and principal investigators, who have to carry out various management and administrative tasks, PhD students are usually free to dedicate their working hours almost exclusively to academic pursuits that they are (or can become) passionate about. This freedom is one of the aspects that make the PhD experience unique, and it should not be overlooked or taken for granted. Unfortunately, not all PhD students benefit from or are aware of such autonomy, but this ought to be an objective for all PhDs.

A PhD does not consist of a number of uneventful years that culminate in a single success. Rather, there are many steps along the way – such as mastering a technique, completing a series of experiments or activities in the field, or finishing the first draft of a manuscript – and the feeling of accomplishment that comes with each completed milestone should be a source of pride to the student.

### Working in a collaborative environment

Learning how to work with other researchers is an important part of getting a PhD. The PhD student’s most important working relationship is with their supervisor (or, in some cases, supervisors), but most PhD students will also have the opportunity to collaborate with other members of their research group or lab, or even with researchers from the wider community. Working on other projects from time to time can help the student’s own project through increased productivity and creativity; moreover, it can strengthen lab cohesion, and might even lead to the student being a co-author on a paper. Additionally, supervising undergraduate students – or even new graduate students – is a good way of acquiring management skills.

Conferences are another way to meet and interact with other researchers. In particular, they are an opportunity to discover, discuss and be inspired by the work of other scientists. Conversations at conferences can generate new research questions or ideas for new and improved ways to tackle existing questions. Moreover, presenting results at a conference gives students a chance to receive feedback, to be recognized as active researchers by their peers, and to build a professional network.

Collaboration also can happen through the many virtual communities that PhD students can join for technical, scientific or moral support. For example, the Global PhD Server enables doctoral students to discuss their experience, exchange anecdotes, and offer or seek help. The @PhDForum supports a variety of activities, such as writing sessions for PhD students working on papers or chapters of their thesis, while Stack Overflow is a good place to offer/seek help with coding and statistics.

### Developing communication skills

The ability to communicate results is a crucial skill for any researcher. A PhD student will, for example, be required to present their work to other scientists as talks or posters at meetings and conferences. The student will also start learning how to write a scientific article. Moreover, there are many opportunities for PhD students to share their passion and knowledge about their field, such as teaching and mentoring undergraduates and other graduate students. They can also get involved in public outreach, and contribute to awakening new passions or educating citizens on certain topics.

## Recommendations

Along the PhD journey, neither the doctoral student nor the supervisor will have full control over what will happen. Some things will go wrong, which is why it is important to remain positive and try to make the most of what is a unique opportunity. Ways for the student to remain positive include going back to old pages in their laboratory notebook to see how much progress has been made, and keeping a note of all the positive feedback from different people. It is also important to remember that one does not become a PhD student by chance – being accepted to do a PhD is an achievement in itself. Additionally, sharing preliminary results with other members of the group and attending social events of a lab can build a supportive working atmosphere and help students to stay positive.

Focusing only on research can sometimes be exhausting, so spending time on other activities – such as supervising students, teaching, or working on outreach – can break the monotony and generate a sense of progress. Finally, it is important to celebrate achievements, such as a first draft, an accepted paper, a conference presentation or the submission of a grant proposal (and, obviously, a successful grant proposal). These achievements can be celebrated in the real world, on social media – or both! By regularly highlighting positive outcomes, it is easier to recognise that past difficulties have been overcome, that progress has been made, and that expertise, skills and knowledge have been gained.

In parallel, it is important to try to limit the impact of the negative aspects of the PhD experience, for they are real and various, and can be crushing if left unchecked. First, it is essential to contextualize them. For example, bear in mind that failure is an integral part of progress, and is often just a temporary setback as opposed to a defeat. This is especially true when a manuscript is rejected by a journal: viewing the rejection as an opportunity to improve the manuscript, and acknowledging that the reviewer reports are about the science, not the authors, can help reframe rejections in a positive light. After all, even the most distinguished researchers have experienced rejection many times. Moreover, as highlighted above, science is a collective adventure, and one is rarely alone when help is sought out. In this regard, talking about the challenges one encounters during a PhD with other students or researchers can also help put these challenges into perspective and to see the positive aspects.

The relationship between the PhD student and their supervisor will likely have a big influence on the PhD experience. However, it is important to recognize that this relationship works both ways, and both stand to benefit if it works well. Among other things, the PhD students can help their own cause by being clear on the type of feedback they want, or by scheduling regular meetings focused on their PhD – and persisting even if their supervisor is busy (Kearns and Gardiner, 2011).

We would also encourage supervisors to be positive in their interactions with their PhD students, and to build a global productive environment that could benefit the PhD student (Andreev et al., 2022). Supervisors could, for example, praise PhD students when the opportunity arises, and ensure that criticism is always constructive – and also encourage other members of their lab to do the same.

PhD students may also face challenges that cannot be overcome with positive thinking. Abusive behaviours such as bullying, harassment or discrimination should be reported to the relevant authorities immediately.

Some PhD students will also be anxious about their future job prospects, especially if they hope to remain in academic research. One way to help reduce such anxiety is to clarify life/career goals and identify the steps needed to reach them. For example, if the student makes a list of all potential funding opportunities (including deadlines) at the start of their last year, it will help them plan for the future and relieve some of the pressure that will build up towards the end of their PhD. Building a professional network can also help with career planning, and attending conferences and establishing collaborations are crucial in this regard.

Finally, if needed, it is entirely acceptable for a PhD student to take a break during their PhD, to refocus on what they really want in life, or to even leave their PhD without finishing it if they realize that it is not for them. However, before making such a decision, we would encourage the student to ask themselves if the doubts they are experiencing are due to a momentary difficulty that will pass, or if a PhD is not really the right career path for them.

Doing a PhD is a unique experience that typically occupies three or more years of someone’s life. Through this experience the student will be enriched by acquiring a range of professional and personal skills, and by gaining a prestigious qualification. In the end, it is in the interest of everyone – the PhD student, the supervisor, their colleagues, their institutions, and academia in general – to make this experience as positive as possible.

## Data Availability

All data generated or analysed during this study came from Twitter API and cannot be shared.
